# Effectiveness of Single‐Wavelength 755 nm Laser Compared to Dual‐Wavelength 755 nm and 1064 nm Laser in Hair Removal: A Split‐Body Design With Blinding

**DOI:** 10.1002/hsr2.72129

**Published:** 2026-03-30

**Authors:** Kawthar Shurrab, Roaa Munif Alnajjar

**Affiliations:** ^1^ Biomedical Photonics Laboratory, Higher Institute for Laser Research and Applications Damascus University Damascus Syrian Arab Republic

**Keywords:** alexandrite laser, efficacy, laser hair removal, Nd:YAG laser, safety

## Abstract

**Introduction:**

Laser hair removal (LHR) has become a popular cosmetic procedure for long‐term hair reduction, with various laser wavelengths and technologies available.

**Objective:**

This study aimed to compare the safety and effectiveness of single‐wavelength 755 nm laser compared to dual‐wavelength 755 nm and 1064 nm laser in hair removal.

**Methods:**

Twenty Syrian women, aged 20–33, with complete data, underwent three axillae laser hair removal treatments at 4‐week intervals between January 2024 and May 2024. The participants, who had Fitzpatrick skin types II and III, were divided into two groups using a split‐body design with blinding, resulting in a total of 40 axillae treated. They were treated with the Duetto MT laser system, which utilizes Alexandrite and Nd: YAG lasers emitting at 755 nm and 1064 nm, individually or in combination, and equipped with a 14 mm Spot Handpiece. Efficacy was assessed using standardized photographs, hair counts, and the Global Aesthetic Improvement Scale (GAIS). Pain was evaluated using a Visual Analog Scale (VAS), and safety outcomes were recorded.

**Results:**

A superior outcome was recorded for a single‐wavelength laser, which showed an overall hair reduction of 92% compared to the dual‐wavelength laser an overall reduction of 69%. Hair counting showed a 25% greater hair reduction with a single wavelength. No severe adverse effects were observed or reported for both groups.

**Conclusion:**

For skin types II and III, the 755 nm laser is more effective for axillary hair removal than the dual‐wavelength laser, even 12 months after treatment. The procedure is considered safe. In contrast, dual‐wavelength laser therapy offered no extra benefits and caused slightly more adverse effects.

## 1 Introduction

Unwanted hair growth is a troubling aesthetic issue for both men and women. It can cause significant psychological distress, impacting overall quality of life and self‐confidence [1, 2]. Although various methods including tweezing, waxing, shaving, chemical hair removal, and electrolysis have been tried to address this problem, they offer only temporary relief. They are sometimes associated with dermatological complications such as folliculitis, burns, contact dermatitis, and post‐inflammatory hyperpigmentation [3]. In comparison, several clinical studies have demonstrated that laser hair removal is a safer option, with fewer adverse effects and longer‐lasting outcomes [3, 4, 5]. Moreover, its safety and efficacy have been reinforced by the approval of the United States Food and Drug Administration (FDA) for long‐term hair reduction since the late 1990s, further supporting its widespread clinical acceptance [6, 7]. Laser hair removal, also known as selective photo‐thermolysis, involves targeting the melanin in hair follicles to destroy them while minimizing damage to surrounding tissues [5]. There are three primary mechanisms by which light can destroy hair follicles: photothermal destruction, photomechanical destruction, and photochemical destruction [5].

Since the late 1990s, following the advent of the Alexandrite solid‐state laser, there have been continuous developments and enhancements in devices and equipment for effective and safe hair removal across all skin types. Initially, the focus was on improving hair removal techniques for individuals with higher melanin content (darker skin), as early lasers were limited in their versatility. These lasers emitted single and short wavelengths (755 nm and 810 nm), making them effective for subjects with lighter skin but not for those with higher melanin content [6, 7]. Nowadays, the leading devices on the market for photo‐epilation have wavelengths of 755, 810, and 1064 nm [8, 9].

LHR is a widely favored cosmetic procedure that typically requires monthly treatments and multiple sessions [10]. While the best candidates are those with fair skin and thick, dark hair, the procedure can be effectively performed on all Fitzpatrick skin types [5, 11].

The outcomes of hair removal treatments can manifest as either complete hair elimination or a reduction in the density or diameter of hair follicles. These results are influenced by various factors affecting the hair growth cycle, such as the location of hair growth, sex, age, and the time of year. Additionally, genetic predisposition, fetal development, medications, and hormonal, nutritional, and psychological factors can also impact hair growth. An accurate initial diagnosis is crucial for achieving optimal results [12, 13].

The results are influenced by various laser parameters such as wavelength, emission type, pulse duration, thermal relaxation time (TRT), pulse frequency, fluence (J/cm²), irradiance (W/cm²), and the spot size of the irradiation beam at the application point [14].

In modern practice, different types of lasers are combined to improve effectiveness and reduce side effects. For example, a new combination of a single multiplex diode laser handpiece that utilizes the benefits of the three most effective wavelengths for hair removal: ALEX 755 nm, SPEED 810 nm, and YAG 1064 nm [15, 16].

Several studies have found that alexandrite and diode laser systems are more effective for hair removal compared to other laser or light‐based devices. One study reported a mean hair reduction of 59.5% for diode laser, 70.3% for alexandrite laser, and 47.4% for Nd:YAG laser after three sessions. Another study showed a mean reduction in hair count of 46% for alexandrite and 27% for intense pulsed light systems. Additionally, in a comparative study, the long pulsed Nd:YAG laser was found to be more effective than the intense pulsed light system for hair removal, with fewer side effects, especially in darker skin types [17, 18, 19].

Previous studies have examined the effectiveness of lasers for hair removal; however, there have been limited direct comparisons between single‐wavelength and dual‐wavelength lasers specifically for this purpose. This study aims to fill that gap by investigating the effectiveness of a single‐wavelength 755 nm laser compared to a dual‐wavelength 755 nm and 1064 nm laser in axillary hair removal in skin types II and III. The study will evaluate the efficacy, pain, and side effects of both methods.

## 2 Methods and Materials

This was a randomized, right‐left comparison, assessor‐blind clinical study conducted at a medical clinical private center. Before data collection, participants were provided information about the complete treatment protocol and signed an informed consent form.

### 2.1 Clinical Data

Twenty Syrian female participants were enrolled from the Private Medical Center between January 1, 2024 and May 31, 2024. None of the participants had previously received laser treatments in the axilla area. The inclusion criteria required participants to be between 20 and 33 years old, with a mean age of 28.4 ± 3.5 years. The participants were classified as Fitzpatrick skin types II and III, and all treated hair was identified as dark terminal hairs. Interested participants were screened to ensure they met the inclusion criteria.

A thorough examination and a case history were recorded on a card. Female individuals who met the inclusion criteria for participation needed, to be in good health, have unwanted hair in the axilla area, be first‐time laser participants with no previous laser treatments in the axilla area, not be suntanned, have no contraindications to laser, and be willing, committed, and agree to the pre‐and post‐care procedures required for the treatment.

Patients excluded from the study were those who had received any systemic or topical medication, had a concurrent diagnosis of another skin condition or malignancy, had open ulcers or infections on any skin site, had a sensitivity to the light, and were pregnant or breastfeeding.

Females were chosen for the study to focus on one gender and avoid the complexities of hormonal differences between genders. The age group of 20 was selected because, at this age, the body has stabilized after the hormonal fluctuations of adolescence. By the age of 30, the body starts to undergo minor hormonal changes, and beyond that age, hormonal changes become more significant [20].

### 2.2 Split‐Body Design With Blinding

In this study, a split‐body design with blinding was employed. All 20 participants were simultaneously assigned to two groups based on body side: Group A for the 20 left axillae and Group B for the 20 right axillae [21, 22]. Each participant, therefore, received both treatments simultaneously, with the left axilla (Group A) consistently treated using the single‐wavelength 755 nm Alexandrite laser, and the right axilla (Group B) treated using the dual‐wavelength combination of 755 nm Alexandrite and 1064 nm Nd: YAG lasers. Patients and outcome assessors were blinded to the allocation, while only the treating operator was aware of the side assignment in order to correctly adjust device settings. The Duetto m.T. laser system, with Quanta Mixed Technology, the system is based on Alexandrite and Nd: YAG lasers, 755 nm and 1064 nm, in single or combined, In the dual‐wavelength mode, the system delivered Alexandrite (755 nm) and Nd: YAG (1064 nm) pulses sequentially with an inter‐pulse delay in the millisecond range, effectively functioning as quasi‐simultaneous emission. Equipped with a 14 mm spot handpiece, with a separated skin cryo handpiece adapter, this adapter works with an external air‐cooling unit to provide a cold air flow, further minimizing pain and thermal damage. The parameters were set at a frequency of 1.5 Hz and a pulse duration of 7 ms. Participants received a total of three sessions, at intervals of 4 weeks. During the treatment, no topical anesthetics or medications were used, and patients did not experience any discomfort or redness. No specific post‐treatment care recommendations were provided, but patients were advised to use broad‐spectrum sunscreen and avoid hot and humid conditions, sweating, friction, rubbing, cosmetics, and salon procedures for 3–5 days after the laser session. The treatments were administered by a single technician.

Receiving treatment stopped after three sessions, and follow‐up was done for 12 months after the last treatment session. They were divided into two groups:

Group A: Received a single‐wavelength treatment with a 755 nm Alexandrite laser on the left axillary area, at a dose of 16–18 J/cm² per session for one pass.

Group B: Received a dual‐wavelength treatment with a 755 nm Alexandrite laser and a 1064 nm Nd: YAG laser on the right axillary area, at doses of 8–9 J/cm² for the Alexandrite laser and 16–18 J/cm² for the Nd: YAG laser per session for one pass.

These parameters were selected according to the manufacturer's recommended protocol and routine clinical practice for Fitzpatrick skin types II and III. In dual mode, the Nd:YAG laser energy density was set to approximately twice the Alexandrite laser energy density, reflecting the standard operating ratio for this system. Blinding was maintained throughout the study, as neither the participants, the staff, nor the statistician knew which side received which treatment until after the statistical analyses were completed. This ensured that the allocation to the treatment groups was concealed, reducing bias and enhancing the reliability of the results [23].

### 2.3 Laser Treatments

Hairs on the underarms were shaved with a razor 3 days before the treatment. The left side was treated with a 755‐nm alexandrite laser, while the right side received a combination of 755‐nm and 1064‐nm lasers. The treatment parameters were: a 14 mm spot size, 7 ms pulse duration, and 1.5 Hz frequency, with one pass per session. The dose varied according to the sessions as shown in Table 1. The subsequent treatment doses were adjusted based on the skin and hair follicle response, with each participant receiving three treatments at 4‐week intervals.

**Table 1:** Treatment parameters for skin type II and III.

No. session	Treatment parameters			
	Fluence J/cm^2^		Frequency Hz	Pulse duration ms
	Alex	Alex, and Nd: YAG		
First and second	16	8–16	1.5	7
Third and fourth	17	9–17	1.5	7
Fifth	18	9–18	1.5	7

### 2.4 Evaluation and Outcome Measures

Standardized clinical photographs were obtained at baseline, 1 month after the final treatment, and again at the 12 months follow‐up to document changes in hair density and thickness [24, 25, 26, 27]. A mobile device was used for image capture, and follicular counts were quantified with HowMany AI, an automated object‐counting tool whose accuracy was verified against manual counts (HowMany AI, 2024) [28, 29, 30].

Pain perception during the laser hair removal sessions was recorded using a 10‐cm Visual Analog Scale (VAS). Scores were categorized as no pain (0), mild (1–3), moderate (4–6), and severe (7–10) [31, 32].

Following completion of treatment, patients completed a satisfaction questionnaire assessing both the degree of improvement and the presence of adverse effects. Responses were grouped into four categories: mild (< 25% improvement), moderate (25–< 50%), good (50–< 75%), and very good (≥ 75%) [33].

### 2.5 Data Analysis

The data analysis was performed using SPSS version 26.0. Quantitative variables were presented as mean ± standard deviation (SD). We used a paired *t*‐test to compare differences within groups and an independent *t*‐test to assess variations between the two groups. For categorical data, we used a *χ* ^2^ test. A *p* value of less than 0.05 was considered statistically significant. To evaluate the difference between the two technologies, we compared the percentage of hair reduction achieved with the dual‐wavelength laser to that obtained with the single‐wavelength laser applicator using the following equation [22]:

Hair reduction percentage=(dual−wavelength%−single−wavelength%)/single−wavelength%

## 3 Results

### 3.1 Assessment Scores

A group of 20 Syrian female patients, aged between 20 and 33 years with a mean age of 28.4 ± 3.5 years, participated in the study. According to the Fitzpatrick skin type classification, 14 patients (65%) were classified as skin phototype II, while 7 patients (35%) were classified as skin phototype III. The initial fluence parameters were set at 16 J/cm² for the single 755 nm wavelength and 8–16 J/cm² for the dual wavelengths (755 and 1046 nm), with gradual increases as detailed in Table 1.

**Figure 1:**
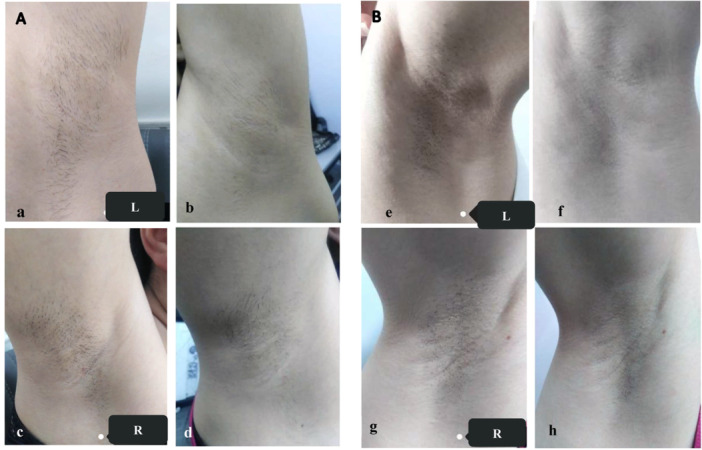
Clinical photographs taken at baseline and 1 month after the final session of three treatments. (A) a 27‐year‐old female with skin phototype II at baseline (a, c) and after three treatments (b, d). The left axilla was treated with a 755 nm laser (b), while the right axilla received dual‐wavelength treatment (d). (B) a 31‐year‐old female with skin phototype III at baseline (e, g) and after three treatments (f, h). The left axilla was treated with a 755 nm laser (f), and the right axilla received dual‐wavelength treatment (h).

The findings reveal the outcomes for the left and right axillae of 20 patients at baseline and 1 month after the final session of three treatments, indicating significant hair reduction in both groups. Both groups showed statistically significant differences (*p *< 0.05). Sample photographs of the left and right axillae of two selected patients at baseline and after three treatments are included in Figure 1 and Table 2. Figure 1A depicts a 27‐year‐old participant, showing significant hair reduction in the left axilla in Group A compared to the right axilla, which was treated in Group B.

**Table 2:** Both groups exhibited statistically significant differences (*p *< 0.05) in the paired samples statistics within groups.

		Mean	*N*	Std. deviation	df	*t*‐value	Sig. difference
Group A	Pre‐(single‐wavelength)	281.80	20	5.14	19	220.82	< 0.05
	After three treatments	24.05	20	2.98			
Group B	Pre‐(dual‐wavelength)	286.80	20	4.91	19	107.47	< 0.05
	After three treatments	84.60	20	5.71			

Figure 1B presents a 31‐year‐old participant with a noticeable decrease in hair density in the left axilla compared to the right axilla.

The comparison between the two groups revealed a significant decrease in hair counts post‐treatment. In Group A, the average hair count reduced from (*M* = 281.80, SD = 5.14) hairs at baseline to (*M *= 24.05, SD = 2.98) after three treatments, (*t* (19) = 220.82, *p *< 0.05). This indicates an average difference of (*M *= 257.75, SD = 5.22), reflecting a mean reduction of 92%. Similarly, Group B experienced a reduction from (*M *= 286.0, SD = 4.91) hairs at baseline to (*M *= 84.60, SD = 5.71), (*t *(19) = 107.47, *p *< 0.05). The average difference was (*M *= 202.20, SD = 8.41), (*t* (38) = 25.08, *p *= 0.048), with a mean decrease of 69%.

**Figure 2:**
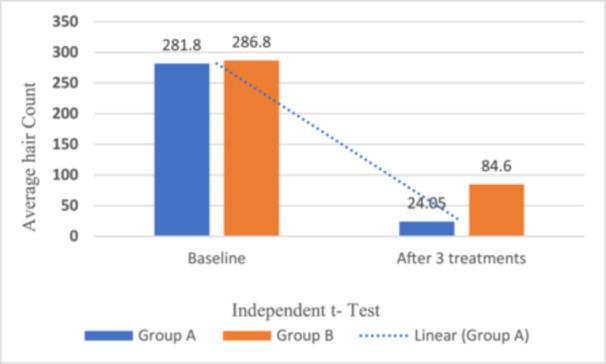
Comparative independent samples test between Group A and B, (92% vs. 69%, with a difference of 25%).

This suggests that the single‐wavelength treatment resulted in a 25% greater reduction in hair than the dual‐wavelength treatment (*p *= 0.048). Overall, in all 20 treated subjects, greater hair reduction was observed in the axillary region treated with the single‐wavelength laser, as presented in Table 3 and Figure 2, which show the average hair counts and GAIS results.

**Table 3:** Independent sample test between Group A and B (*p *= 0.048).

Difference	Group	*N*	Means difference	Std. deviation	Hair reduction rate %	GAIS scale	df	*t*	Sig. difference
	Group A	20	257.750	5.22	92%	5	38	25.08	0.048
	Group B	20	202.20	8.41	69%	4	38	25.08	

The GAIS assessment scores ranged from 4 to 5 for the 755 nm wavelength laser and from 3 to 5 for the dual‐wavelength laser, indicating an improvement of 25% for the side treated with the 755 nm wavelength. On average, the single‐wavelength laser achieved a GAIS score of (*M* = 4.97, SD = 0.52) out of 5, while the dual‐wavelength laser scored (*M *= 3.89, SD = 0.75) out of 5. The 755 nm laser showed a 24% improvement (SD = 12%; *p *= 0.045).

The results confirm the effectiveness and safety of using the 755 nm laser alone or combined with the 1064 nm laser. No burns, irritation, redness, localized infection, skin pigmentation changes, or textural alterations were observed. No significant adverse events were recorded at any follow‐up, including 3‐ and 6‐months post‐treatment. All 20 subjects completed the study visits, including the 6‐month follow‐up.

To the best of our knowledge, this study is the first to evaluate and compare the efficacy of single‐wavelength versus dual‐wavelength laser treatments for underarm hair removal on the same individual using a split‐body design. Additionally, it aims to determine the optimal dose, number of sessions, and follow‐up period over 6 months.

### 3.2 Side Effects and Patient Satisfaction

Patient satisfaction in our study was high in both groups. Six patients (15%) reported their improvement as good, and all other patients (85%) as very good. There was a significant reduction in hair counts for all patients who participated in this study after just three sessions, and the results remained consistent for 12 months later.

**Figure 3:**
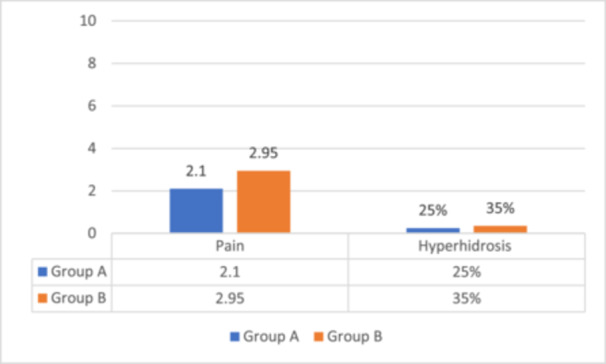
Side effect assessments (pain and axillary hyperhidrosis).

The pain reported by patients during the treatments was comparable in both procedures, with mild pain levels ranging between 2 and 3 during the initial treatment, which subsequently decreased in later sessions. Axillary hyperhidrosis was observed following LHR in both groups, with approximately 5 patients (25%) in Group A and a more pronounced occurrence in the area treated with the dual‐wavelength, affecting 7 patients (35%) in Group B, as illustrated in Figure 3. Some patients experienced spontaneous recovery of normal axillary transpiration, while others continued to experience hyperhidrosis up to 6 months post‐treatment.

These effects were moderate across all treated subjects. No unexpected or severe significant adverse events were recorded at any of the follow‐up time points, 3 and 6 months post‐last treatment. Additionally, no instances of burns, hyperpigmentation, depigmentation, or paradoxical hair growth were reported on either side.

## 4 Discussion

Laser hair removal is a widely sought cosmetic procedure, particularly among women, driven by aesthetic preferences, personal comfort, and hygiene considerations [33, 34]. Although numerous studies have investigated lasers with different wavelengths and the introduction of multi‐wavelength techniques, many of these studies are limited, often evaluating different skin types in separate individuals [22, 35]. To our knowledge, this is among the first studies to directly compare the efficacy of single‐wavelength and dual‐wavelength laser treatments for axillary hair removal within the same individual using a split‐body design. Furthermore, it provides clinically relevant data regarding treatment parameters and long‐term outcomes over a 12‐month follow‐up period in patients with Fitzpatrick skin types II–III.

The single‐wavelength 755 nm laser demonstrated a mean hair reduction of 92%, significantly outperforming the dual‐wavelength laser, which achieved a 69% reduction. This 25% greater reduction in hair counts with the single‐wavelength laser highlights its superior efficacy. The superior performance of the 755 nm single‐wavelength laser observed in this study is attributable to its stronger absorption by melanin, enabling more precise and efficient follicular destruction. In contrast, dual‐wavelength therapy is intended to balance epidermal protection with follicular heating: the 1064 nm Nd: YAG penetrates more deeply with minimal melanin absorption, thereby lowering the risk of epidermal damage, while the 755 nm Alexandrite more directly targets the melanin‐rich hair shaft. This rationale may be advantageous in challenging scenarios, such as patients with lighter hair on darker skin or higher Fitzpatrick types, where safety and treatment efficacy are more difficult to achieve. However, in our cohort of Fitzpatrick II–III patients with dark terminal hair, the Alexandrite wavelength alone provided superior efficacy and comparable safety. These findings are consistent with theoretical models of selective photothermolysis, which predict that Alexandrite would outperform a dual‐mode approach in lighter skin due to its higher melanin specificity. Importantly, our results not only align with these predictions but also confirm them clinically under controlled conditions, showing that the addition of Nd:YAG energy may dilute the follicular effect rather than enhance it in this patient group.

The treatment parameters were selected in accordance with both the manufacturer's recommended protocol and established clinical practice. In dual‐wavelength mode, the Nd:YAG fluence was set at approximately twice that of the Alexandrite, consistent with the standard operating configuration of this device. This ratio reflects the lower melanin absorption and deeper dermal penetration associated with the 1064 nm wavelength. Accordingly, the reduced Alexandrite fluence in dual mode represents the intended thermal distribution strategy of the system, whereby energy is distributed across different tissue depths rather than concentrated at a single absorption peak. Matching the Alexandrite fluence to the single‐wavelength parameters would have resulted in substantially higher cumulative energy delivery, thereby altering the intended energy balance between the two wavelengths.

Patient‐reported outcomes, including satisfaction and pain, were evaluated using the Global Aesthetic Improvement Scale (GAIS). The single‐wavelength laser achieved an average GAIS score of 4.97 out of 5, compared to 3.89 out of 5 for the dual‐wavelength laser. This indicates not only a higher level of hair reduction but also greater patient satisfaction with the aesthetic outcomes.

Our study demonstrated that treatment with a 755 nm alexandrite laser was relatively safe and did not cause serious or unexpected side effects. Although dual‐wavelength laser techniques have been proven to be safe and effective for laser hair removal (LHR), some patients reported moderate pain during treatment and axillary hyperhidrosis with the dual‐wavelength laser. While some patients experienced spontaneous recovery of normal axillary transpiration, others continued to experience hyperhidrosis for up to 12 months post‐treatment. This study is one of the few that confirms the side effect of sweating under the armpit after LHR. However, the single‐wavelength laser causes fewer instances than the dual‐wavelength laser, even though both techniques are from the same device equipped with a cryogen spray cooling device, which significantly reduces discomfort during treatment.

The outcomes of this study have significant implications for clinical practice. The superior performance of the 755 nm single‐wavelength laser suggests that it may be a more effective option for patients seeking hair removal treatments. Additionally, using a single wavelength simplifies the treatment protocol, potentially reducing treatment time and cost.

It is important to acknowledge the limitations of this study, as it was focused exclusively on patients with Fitzpatrick skin types II and III, which restricts the generalizability of the findings to individuals with darker skin types (IV–VI). In such populations, unique risks such as post‐inflammatory hyperpigmentation and thermal injury must be considered, and the Nd: YAG laser (1064 nm) is often regarded as a safer option because of its deeper penetration and lower melanin absorption. In addition, the relatively small sample size represents another constraint. Future investigations should aim to include larger and more diverse populations, extend the follow‐up period, and incorporate improved clinical documentation to validate the durability and broader applicability of the results observed with the single‐wavelength laser.

## 5 Conclusion

This pioneering study shows that the single‐wavelength 755 nm laser is significantly more effective for axillary hair removal in women compared to the dual‐wavelength laser. It achieved a 25% greater reduction in hair counts, along with slightly higher patient satisfaction scores and fewer side effects. These factors make it a promising option for hair removal treatments. Further research is necessary to evaluate the effectiveness of single and dual wavelengths on other body areas and skin types.

## Author Contributions

**Kawthar Shurrab:** conceptualization, investigation, writing – original draft, methodology, validation, visualization, writing – review and editing, software, formal analysis, project administration, data curation, supervision, and resources. **Roaa Munif Alnajjar:** investigation and resources.

## Funding

The authors have nothing to report.

## Ethics Statement

The ethics committee of Damascus University approved the study ID number: HILRA‐051124‐348. Written informed consent was obtained for the publication and the use of all patients' images before their enrollment in the study. This study was performed by the Helsinki Declaration of 1964 and its subsequent amendment.

## Conflicts of Interest

The authors declare no conflicts of interest.

## Transparency Statement

The lead author, Kawthar Shurrab, affirms that this manuscript is an honest, accurate, and transparent account of the study being reported; that no important aspects of the study have been omitted; and that any discrepancies from the study as planned (and, if relevant, registered) have been explained.

## Data Availability

All data are included in the manuscript.
